# Event and Comment

**Published:** 1917-04

**Authors:** 


### DENTAL REGISTER

Vol. LXXI APRIL, 1917 No. 4

### EVENT AND COMMENT

THE DENTAL SOCIETY HABIT. In almost every department of human endeavor we find most of the activities are influenced by what we might term the habitual. We are influenced at times to do the unconventional from caprice, or because we have a larger vision of our environment. But we are Democrats or Methodists because father and mother embraced these faiths. We even attend church sometimes because of an early formed impression that this is the proper form of life rather than for the nobler demands of our natures. It is a curious fact that some dentists are always present at their local or State society meetings, whilst others are wholly indifferent and attend only when the spirit moves or there is something to be had. It was said of Dr. A. F. Emminger that he had not missed a meeting of the Ohio State Dental Society in the forty-nine years he has been a member. That is some record, and perhaps it may not be wholly due to the force of habit. Emminger was a devoted member of the profession, and his loyalty had a deeper meaning.

But the fact is that most dentists attend dental meetings because they have the habit strongly fixed in their minds. Whether they have anything to contribute or hope to get anything or not when the time comes for the meeting, you find they get anxious and can't stay at home. Now this applies, of course, only to the habitual attendant. By far the larger class of dentists are not worried by the habit, and they feel no compulsion to contribute to the program, or even by their presence. If it is convenient and business is not too pressing, they will go, or if the program seems attractive they may make a special effort to be there. Our program committees believe in the drawing power of live programs, with star performers and unusual stunts, and in this way they bait us in. The trouble with this method is that it has limitations, and it is not always possible to offer a star performance, and then there is a dull meeting, and a collapse that discredits the society in the estimation of the thoughtless for several years.

It has become common practice to make our large society forums the field for the exploitation of the new or novel and the extra skillful artist. So there is no place there for the cultivation of the society habit by the modest man, who fears to show his little torch amid so much brilliancy. When the reorganization plan was adopted a few years ago, it was hoped that the district societies would do much to develop latent talent and supply talent to the State meetings, and this has proved true in some cases. It has developed that in the small societies where meetings are held more frequently there has been more general participation by all the members, and consequently greater enthusiasm for the cause. Curiously enough, it has been found in Illinois that more members, relatively, attend the State meeting from the district societies which held their meetings at the most frequent intervals. While 50 percent of the members of district societies holding nine meetings each year attend the State meeting, only 32 percent of the members of the local societies, which hold only one meeting per year, attend the annual State meeting.

Corresponding data can be cited from other States. The explanation is that the habit of attending so many meetings each year and the constant giving and getting in the local meetings creates an appetite which urges one to seek for further and better knowledge elsewhere. Is there not something here that the managers of professional activities should consider more carefully, and, if the idea is correct, should they not adopt some measures that may lead to more general attendance of the local society meetings by the indifferent members, with the purpose of inculcating the spirit of seeking after more knowledge, and, incidentally, of forming a habit which may enhance the general knowledge and development of our profession?

In small places, where no component society exists, it would be most helpful to organize subsidiary clubs which would hold frequent and informal meetings. Much benefit would come to those attending and a professional spirit would be engendered that would be well worth while. It would in this connection be worth while to read in *Oral Hygiene* for April, page 387, an account of the Hartford (Conn.) Dental Society that meets every Monday for nine months of the year, with an average attendance of thirty-five members.

CONSOLIDATION OF LICENSING BOARDS. The Illinois Legislature is considering a law that will consolidate the various licensing boards under a single administrative office. Graduates of medical, dental and pharmacal schools will be examined as before by their respective examining boards, but these boards are to be appointed and report to the officer or director of the department of registration and education. This plan may operate to economical advantage in the way of lessening the number of licensing bodies, and it should, if wisely conducted, become a more efficient method of standardizing qualifications for professional practice. Whether it is wise to place so important a function in the hands of a department having little or no personal relations to the various callings affected is a matter of some doubtful propriety. If the head of the department should be wise, honest and efficient, he will take a personal interest in the business of his department, and there can be little doubt as to the wisdom of the scheme. We have for a long time contended that all licensing of persons having to do with the public health should be concentrated into an efficiently organized State health bureau. We believe in this way better health laws would be possible and stricter enforcement of law would result, to the great advantage of the people.

